# Community pharmacists’ knowledge, attitudes, and educational needs regarding LGBTQ+ healthcare: a cross-sectional study

**DOI:** 10.1080/20523211.2026.2753953

**Published:** 2026-11-04

**Authors:** Michael Petrides, Ilias Tanis, Vasileios Kiosses, Aliki Peletidi

**Affiliations:** a Pharmacy Programme, Department of Health Sciences, School of Life and Health Sciences, University of Nicosia, Nicosia, Cyprus; b Bioactive Molecules Research Center, School of Life and Health Sciences, University of Nicosia, Nicosia, Cyprus; c Psychotherapist- Medical Educator, Athens, Greece; d Department of Health Sciences, School of Life and Health Sciences, University of Nicosia, UNIC Athens, Athens, Greece

**Keywords:** LGBTQ+ health, community pharmacists, cultural competence, pharmacy education, inclusive healthcare, health equity

## Abstract

**Background:**

LGBTQ+ individuals continue to experience persistent healthcare inequalities, partly due to limited training of healthcare professionals in LGBTQ+-specific healthcare needs. Community pharmacists (CPs), given their accessibility and role in frontline healthcare delivery, are well-positioned to provide inclusive and competent care. This study aimed to describe participating CPs’ self-reported knowledge and awareness, attitudes, professional experiences, educational exposure, and educational needs regarding LGBTQ+ healthcare, and to explore whether responses to selected items on difficulty providing care and participation in LGBTQ+ advocacy differed by demographic and professional characteristics.

**Methods:**

We conducted a quantitative cross-sectional study in Thessaloniki, Greece, between October 2023 and January 2024, involving 305 CPs (59.8% of the 510 pharmacists invited). Data were collected using a 35-item questionnaire covering self-reported knowledge and awareness, educational exposure and preparedness, ethical perceptions, professional experiences and behaviours, advocacy and awareness activities, and demographic and professional characteristics. Descriptive statistics and unadjusted subgroup comparisons were performed using SPSS, applying independent-samples t-tests and one-way ANOVA, as appropriate. Statistical significance was set at *p* < 0.05.

**Results:**

Participants reported generally positive attitudes toward LGBTQ+ patients; however, only 43.0% reported adequate training in LGBTQ+-specific healthcare needs. Participation in LGBTQ+ advocacy and awareness activities differed significantly across age groups (F(3,301) = 7.058, *p* < 0.001), with younger pharmacists reporting higher engagement. Advocacy participation also differed significantly across professional-experience groups (F(2,302) = 9.146, *p* < 0.001), with pharmacists with fewer than 10 years of experience reporting greater involvement than those with >10 years. Female pharmacists reported significantly higher advocacy engagement than males (t(303) = −4.089, *p* < 0.001). No robust statistically significant group differences were identified in self-reported difficulties in providing care across the demographic and professional characteristics examined.

**Conclusions:**

While attitudes are positive, important educational gaps persist. This highlights the need for structured LGBTQ+ inclusive undergraduate pharmacy curricula and continuing professional development to ensure competent, respectful, and inclusive care for LGBTQ+ individuals.

## Background

LGBTQ+ individuals worldwide experience persistent healthcare inequalities driven by stigma, discrimination, social marginalisation, and inadequate provider training (Grundmann et al., 2020; Moagi et al., 2021; Morris et al., 2019). These inequities contribute to poorer health outcomes, including higher rates of mental health disorders, suicide attempts, substance use, HIV infection, and certain cancers (CDC, 2019; Fradelos et al., 2022; Grundmann et al., 2020). Fear of discrimination frequently leads to avoidance of healthcare services, further exacerbating disparities (ILGA-Europe, 2020; UNDP-PGA, 2022).

Healthcare professionals, including pharmacists, are well positioned to address these challenges through culturally competent care (Arthur et al., 2021; Heredia et al., 2021). Community pharmacists, as highly accessible healthcare providers (Community Pharmacy at a Glance 2021: Regulation, scope of practice, remuneration, and distribution of medicines through community pharmacy premises and other outlets (2021) – FIP Development Goals, n.d.), can contribute to sexual health promotion, HIV prevention, gender-affirming care, counselling, and mental health support (Furness et al., 2020; Grundmann et al., 2020; Wahlen et al., 2020). However, many pharmacists report limited knowledge, confidence, and formal training in LGBTQ+-specific healthcare, reflecting persistent gaps in undergraduate curricula, postgraduate education, and continuing professional development (Arthur et al., 2021; Furness et al., 2020; Heredia et al., 2021; Morris et al., 2019; Wahlen et al., 2020).

Evidence from Greece and Southern Europe remains scarce, with most research originating from North America and Western Europe (Arthur et al., 2021; Fradelos et al., 2022; ILGA-Europe, 2020). Cultural, educational, and healthcare system factors unique to Greece may influence pharmacists’ readiness to deliver LGBTQ+-inclusive care (Pagkozidis et al., 2025; Peletidi et al., 2025). This study aligns with the International Pharmaceutical Federation (FIP) Development Goals, particularly Goal 10 (Equity and Equality) and Goal 8 (Working with Others), by addressing workforce readiness for inclusive, patient-centred care.

The aim of this study was to describe participating community pharmacists’ self-reported knowledge and awareness, attitudes, professional experiences, educational exposure, and educational needs regarding LGBTQ+ healthcare, and to explore whether responses to selected items concerning difficulty in providing care and participation in LGBTQ+ advocacy differed according to demographic and professional characteristics. The study is guided by Minority Stress Theory, which highlights the cumulative impact of stigma and discrimination on LGBTQ+ health disparities (Valentine & Shipherd, 2018).

## Methods

### Study design and reporting

This cross-sectional study employed a quantitative survey design and was reported in accordance with the STROBE (Strengthening the Reporting of Observational Studies in Epidemiology) guidelines for cross-sectional studies to ensure methodological rigour and transparency (von Elm et al., 2007).

### Setting, participants, sampling, and sample size

The study was conducted in Thessaloniki, Greece, between October 2023 and January 2024. All participants were practising in community pharmacies within the Municipality of Thessaloniki; pharmacists working in hospitals, primary care centres, or other healthcare settings were not included. Through the regional pharmacists’ association and the available recruitment channels, 510 individual practising community pharmacists were contacted and invited to participate. Both community pharmacy owners and employed community pharmacists were eligible.

The minimum sample size was estimated using the Raosoft sample-size calculator (Raosoft, 2026). Because the accessible recruitment pool consisted of 510 pharmacists, a finite-population calculation was applied using a 95% confidence level, a 5% margin of error, and a 50% response distribution. The 50% response distribution was selected as the most conservative assumption because no prior estimate was available for the proportion expected to give a particular response regarding knowledge, attitudes, or another study outcome. Under these assumptions, the finite-population adjusted minimum sample size was 220 participants (Sample Size Calculator by Raosoft, Inc, 2004). The final analytical sample comprised 305 pharmacists, exceeding this minimum and corresponding to 59.8% of the 510 pharmacists invited. For broader context, official data reported 1,192 community pharmacies in the wider Thessaloniki Prefecture in 2023 (Hellenic Statistical Authority (ELSTAT), n.d.). Applying the FIP estimate of 1.05 pharmacists per community pharmacy corresponds to approximately 1,252 pharmacists across the Prefecture; this figure was not used as the sampling denominator for the present study. Achievement of the calculated minimum sample size was not interpreted as establishing representativeness because participation was voluntary and nonresponse or self-selection bias could not be excluded.

### Eligibility criteria

Eligible participants were actively practising community pharmacists registered with the regional pharmacy association of Thessaloniki, holding an active licence and employed in a community pharmacy at the time of data collection. Both pharmacy owners and staff pharmacists were included. Pharmacists with inactive licences or who declined informed consent were excluded.

### Instrument development and validation

The questionnaire was developed following a comprehensive review of the literature on LGBTQ+ healthcare, cultural competence, and pharmacy practice, drawing on prior instruments and studies (Arthur et al., 2021; Furness et al., 2020; Heredia et al., 2021; Wahlen et al., 2020). An expert panel (*n* = 6), comprising academic pharmacists, LGBTQ+ health specialists, a public health researcher, and a practising community pharmacist, independently reviewed the questionnaire for relevance, clarity, cultural appropriateness, and comprehensiveness. All six experts considered the questionnaire acceptable in its original form and did not recommend deletion, addition, rewording, or modification of any item. The review did not use Lawshe’s three-category essentiality classification; therefore, item-level Lawshe content validity ratios were not calculated prospectively and could not be reconstructed reliably from the original expert review.

A pilot study with ten eligible community pharmacists confirmed feasibility and acceptibility; eight participants (80%) reported the questionnaire to be clear and culturally appropriate, while two participants suggested minor terminology refinements. Pilot participants were excluded from the final sample.

The final instrument consisted of 35 structured items organised thematically to assess self-reported LGBTQ+ knowledge and awareness; educational exposure and perceived preparedness; ethical and clinical perceptions; professional experiences and self-reported behaviours; engagement in LGBTQ+ advocacy and awareness activities; and demographic and professional characteristics (Supplemental Survey S1). Items used multiple-choice, frequency-based, and Likert-type response formats. The questionnaire was not designed to generate a single total score because its items addressed heterogeneous constructs. Internal consistency was therefore assessed separately for three conceptually defined item sets. The conceptually defined item set concerning perceived LGBTQ+ education and preparedness (Items 4–6) demonstrated good internal consistency (Cronbach’s α = 0.845), with corrected item–total correlations ranging from 0.693 to 0.729; removal of any item reduced alpha. The two-item set concerning interest in further LGBTQ+ education (Items 7–8) demonstrated very high internal consistency (Cronbach’s α = 0.929; inter-item r = 0.867), and the two-item set concerning LGBTQ+ advocacy and awareness activities (Items 27–28) demonstrated good internal consistency (Cronbach’s α = 0.859; inter-item r = 0.754). These coefficients describe internal consistency within the specified item sets and should not be interpreted as evidence of reliability for the questionnaire as a single overall scale. These internal-consistency analyses were used to characterise the coherence of the specified conceptually related item sets; they do not imply that composite scores were constructed or used in the subgroup analyses.

### Data collection and ethics

Data were collected between October 2023 and January 2024 using electronic (UNIC Moodle platform) and paper-based questionnaires distributed via professional meetings and pharmacy association offices. Participation was voluntary, anonymous, and confidential, with informed consent obtained from all participants. Ethical approval was granted by the Ethics Committee of the Thessaloniki Pharmaceutical Association (Reg. no. THPA-2023-09).

### Outcome measures

The questionnaire assessed participating pharmacists’ self-reported knowledge and awareness of LGBTQ+ terminology and healthcare issues, educational exposure and perceived preparedness, ethical and clinical perceptions, professional experiences and behaviours when providing care, engagement in advocacy and awareness activities, and educational needs. Subgroup analyses focused on selected responses concerning reported difficulty in providing care and participation in LGBTQ+ advocacy and awareness activities.

### Statistical analysis

Data were analysed using IBM SPSS Statistics version 28. Descriptive statistics (frequencies, means, and standard deviations) summarised participant characteristics and questionnaire responses. Unadjusted subgroup comparisons were conducted using independent-samples t-tests for binary demographic/professional factors and one-way ANOVA for factors with more than two groups; Welch-corrected tests were used where appropriate. Two individual ordinal questionnaire items were analysed as subgroup outcomes: Item 22, assessing the frequency of difficulty serving a person whose appearance did not match the details of their identification, and Item 27, assessing the frequency of participation in events or initiatives supporting the rights of LGBTQ+ individuals. Both items used five-point frequency response scales coded from 1 (Never) to 5 (Always); higher scores therefore indicated greater reported difficulty for Item 22 and greater advocacy participation for Item 27. For subgroup analyses, educational level was collapsed to Bachelor versus Postgraduate (Master’s and Doctorate), professional experience to <6 years, 6–10 years, and >10 years, and age to four groups by combining the two oldest categories (53–62 and ≥63 years). Effect sizes were reported as Hedges’ g for t-tests and η² for ANOVA, with 95% confidence intervals where applicable. Statistical significance was set at *p* < 0.05 (two-tailed).

## Results

A total of 305 community pharmacists participated in the study. The sociodemographic profile of participants is presented in Table 1. The gender distribution was balanced, with 157 females (51.5%) and 148 males (48.5%). Regarding age, the largest proportion of participants were aged 23–32 years (29.2%), followed by 33–42 years (27.2%), 43–52 years (23.3%), 53–62 years (13.8%), and 63 years or older (6.6%). In terms of sexual orientation, 0.7% of respondents self-identified as LGBTQ+, while 3.3% preferred not to disclose. The majority of pharmacists held a bachelor’s degree (69.5%), with 27.9% holding postgraduate degrees and 2.6% doctoral degrees. Most respondents were pharmacy owners (55.7%), while 44.3% were employees. Regarding professional experience, 51.1% reported more than 10 years of experience, 17.7% had 6–10 years, 23.9% had 1–5 years, and 7.2% had less than one year.

**Table 1.** Sociodemographic characteristics of participants (*N* = 305).

Characteristic	Category	*N*	%
Age	23–32 years	89	29.2
	33–42 years	83	27.2
	43–52 years	**71**	23.3
	53–62 years	42	13.8
	≥63 years	**20**	6.6
Gender	Male	148	48.5
	Female	157	51.5
LGBTQ+ identification	Yes	2	0.7
	No	293	96.1
	Prefer not to answer	10	3.3
Education	Bachelor’s degree	212	69.5
	Master’s degree	85	27.9
	Doctorate	8	2.6
Employment	Employee	135	44.3
	Owner	170	55.7
Years of experience	<1 year	22	7.2
	1–5 years	73	23.9
	6–10 years	54	17.7
	>10 years	156	51.1

The participants’ knowledge and understanding of LGBTQ+ terminology, healthcare needs, and educational readiness are presented in Table 2. The majority correctly recognised that the LGBTQ+ community refers to individuals with different sexual orientations, gender identities, and gender expressions (93.6% agreement). Similarly, 89.6% stated they understood the distinction between sexual orientation and gender identity.

**Table 2.** Knowledge, awareness, and educational needs (*N* = 305).

Question	Response	N	%
LGBTQ+ includes people with diverse sexual orientation, gender identity and expression	Strongly disagree	2	0.7
	Disagree	6	2.0
	Neither agree nor disagree	11	3.6
	Agree	181	59.3
	Strongly agree	105	34.3
Understand the distinction between sexual orientation and gender identity	Strongly disagree	1	0.3
	Disagree	9	3.0
	Neither agree nor disagree	22	7.2
	Agree	142	46.6
	Strongly agree	131	43.0
Familiarity with LGBTQ+ terminology	Strongly disagree	2	0.7
	Disagree	45	14.8
	Neither agree nor disagree	87	28.5
	Agree	142	46.6
	Strongly agree	29	9.5
Adequate education on LGBTQ+ healthcare needs	Strongly disagree	8	2.6
	Disagree	64	21.0
	Neither agree nor disagree	102	33.4
	Agree	110	36.1
	Strongly agree	21	6.9
Education covered LGBTQ+ discrimination and needs	Strongly disagree	18	5.9
	Disagree	73	23.9
	Neither agree nor disagree	107	35.1
	Agree	93	30.5
	Strongly agree	14	4.6
Fully equipped to support LGBTQ+ patients	Strongly disagree	6	2.0
	Disagree	61	20.0
	Neither agree nor disagree	98	32.1
	Agree	111	36.4
	Strongly agree	29	9.5
Desire more education	Strongly disagree	8	2.6
	Disagree	18	5.9
	Neither agree nor disagree	69	22.6
	Agree	142	46.6
	Strongly agree	68	22.3
Willingness to attend training	Strongly disagree	3	1.0
	Disagree	25	8.2
	Neither agree nor disagree	63	20.7
	Agree	135	44.3
	Strongly agree	79	25.9
Comfort if colleagues knew about LGBTQ+ care	Strongly disagree	2	0.7
	Disagree	4	1.3
	Neither agree nor disagree	42	13.8
	Agree	95	31.1
	Strongly agree	162	53.1

When asked about familiarity with LGBTQ+ terminology such as transgender, queer, and intersex, 56.1% reported sufficient knowledge. In contrast, 43.9% either disagreed or neither agreed nor disagreed, indicating persistent knowledge gaps. Only 43% agreed that they had received adequate training regarding the specific healthcare needs and concerns of LGBTQ+ individuals, whereas 23.6% expressed disagreement and 33.4% neither agreed nor disagreed.

Participants’ responses concerning LGBTQ+ terminology, healthcare needs, and educational readiness are presented descriptively in Table 2.

The participants’ ethical perceptions concerning LGBTQ+ healthcare are summarised in Table 3. The overwhelming majority (80.6%) agreed that protecting the anonymity of LGBTQ+ patients is essential for trust-building. However, conditional attitudes were also identified, with 69.1% agreeing that they have no problem with LGBTQ+ individuals provided they are not ‘provocative’ in their expressions. Perceptions regarding adolescent LGBTQ+ healthcare revealed that only 41.3% agreed that LGBTQ+ and non-LGBTQ+ adolescents have the same health needs, while 39% either disagreed or neither agreed nor disagreed. Furthermore, 55.4% agreed that gay and lesbian individuals have higher rates of anxiety and depression compared to heterosexual individuals.

**Table 3.** Ethical considerations and perceptions regarding LGBTQ+ health (*N* = 305).

Question	Response	N	%
Protection of LGBTQ+ patients’ anonymity is vital	Strongly disagree	1	0.3
	Disagree	9	3.0
	Neither agree nor disagree	55	18.0
	Agree	136	44.6
	Strongly agree	104	34.1
No problem with LGBTQ+ if not provocative	Strongly disagree	11	3.6
	Disagree	27	8.9
	Neither agree nor disagree	56	18.4
	Agree	84	27.5
	Strongly agree	127	41.6
LGBTQ+ adolescents have same health needs	Strongly disagree	25	8.2
	Disagree	94	30.8
	Neither agree nor disagree	60	19.7
	Agree	80	26.2
	Strongly agree	46	15.1
Gay/lesbian individuals have higher anxiety and depression rates	Strongly disagree	3	1.0
	Disagree	28	9.2
	Neither agree nor disagree	105	34.4
	Agree	125	41.0
	Strongly agree	44	14.4
Blood donation restrictions justified	Strongly disagree	39	12.8
	Disagree	93	30.5
	Neither agree nor disagree	74	24.3
	Agree	67	22.0
	Strongly agree	32	10.5
LGBTQ+ at higher risk of HIV	Strongly disagree	22	7.2
	Disagree	79	25.9
	Neither agree nor disagree	74	24.3
	Agree	89	29.2
	Strongly agree	41	13.4

Regarding issues related to HIV, 32.5% supported restrictions on blood donation by gay men, while 42.6% perceived LGBTQ+ individuals as having a higher risk of HIV infection.

Pharmacists’ behaviours and confidence when interacting with LGBTQ+ patients are summarised in Table 4. Most participants reported regularly serving LGBTQ+ patients, with 66.5% indicating they did so ‘often’ or ‘always,’ while only 8.8% reported rare or no contact. The majority (73.4%) reported no discomfort when providing care, whereas 26.6% expressed some level of discomfort.

**Table 4.** Clinical behaviours, comfort levels, workplace environment and interactions (*N* = 305).

Question	Response	*N*	%
Frequency of serving LGBTQ+ patients	Never	8	2.6
	Rarely	19	6.2
	Sometimes	75	24.6
	Often	59	19.3
	Always	144	47.2
Discomfort while serving LGBTQ+ patients	Never	224	73.4
	Rarely	57	18.7
	Sometimes	17	5.6
	Often	6	2.0
	Always	1	0.3
Administrative difficulties with LGBTQ+ patients	Never	148	48.5
	Rarely	96	31.5
	Sometimes	52	17.0
	Often	8	2.6
	Always	1	0.3
Difficulty when appearance differs from ID	Never	200	65.6
	Rarely	67	22.0
	Sometimes	31	10.2
	Often	5	1.6
	Always	2	0.7
Discussion of LGBTQ+ topics at work	Never	81	26.6
	Rarely	105	34.4
	Sometimes	83	27.2
	Often	36	11.8
	Always	0	0.0
Colleagues making jokes	Never	160	52.5
	Rarely	89	29.2
	Sometimes	39	12.8
	Often	12	3.9
	Always	5	1.6
Self-making jokes	Never	271	88.9
	Rarely	18	5.9
	Sometimes	9	3.0
	Often	6	2.0
	Always	1	0.3

Administrative barriers were common. Over half of respondents (51.5%) reported experiencing administrative or procedural difficulties related to identity documentation, and 34.4% reported challenges when a client’s appearance did not match legal identification. Specifically, 48.5% had never encountered such administrative issues, while 51.5% experienced them to varying degrees (rarely: 31.5%; sometimes: 17.0%; often/always: 2.9%). Similarly, 65.6% had never faced difficulties related to appearance–identification mismatches, whereas 34.4% reported such challenges (rarely: 22.0%; sometimes: 10.2%; often/always: 2.3%).

A substantial proportion of participants therefore reported encountering administrative or identification-related difficulties when serving LGBTQ+ patients.

The workplace environment and attitudes toward LGBTQ+-related conversations and humour are shown in Table 4. A total of 26.6% of participants reported never discussing LGBTQ+-related topics at work, while 11.8% discussed them frequently. Colleagues’ occasional use of jokes or comments about LGBTQ+ individuals was observed by 47.5% of participants, but 88.9% reported that they personally never made such jokes.

The pharmacists’ active engagement with LGBTQ+ advocacy, participation in awareness actions, and utilisation of scientific sources are presented in Table 5. Almost half (49.5%) reported no involvement in any LGBTQ+-related advocacy activities. Furthermore, 53.4% had not initiated any awareness-raising actions in their local communities. A limited proportion (5.2%) reported regularly using scientific literature on LGBTQ+ health issues, suggesting a need for greater professional engagement.

**Table 5.** Active engagement and advocacy activities (*N* = 305).

Question	Response	*N*	%
Colleague insulting LGBTQ+ persons	Never	225	73.8
	Rarely	50	16.4
	Sometimes	25	8.2
	Often	4	1.3
	Always	1	0.3
Participation in LGBTQ+ advocacy	Never	151	49.5
	Rarely	85	27.9
	Sometimes	54	17.7
	Often	8	2.6
	Always	7	2.3
Awareness actions in community	Never	163	53.4
	Rarely	77	25.2
	Sometimes	50	16.4
	Often	13	4.4
	Always	2	0.7
Use of LGBTQ+ scientific literature	Never	54	17.7
	Rarely	46	15.1
	Sometimes	119	39.0
	Often	70	23.0
	Always	16	5.2

To compare responses across sociodemographic and professional groups, one-way ANOVA and independent-samples t-tests were performed for the selected reported-difficulty and LGBTQ+ advocacy outcomes. A 95% confidence level was applied to all analyses.

- Group Differences in Reported Difficulties by Demographic and Professional Factors

Multi-level factors (age and professional experience) were compared using one-way ANOVA (Welch-corrected when appropriate), whereas binary factors (education after collapsing and gender) were compared using independent-samples t-tests with Welch corrections where appropriate. Effect sizes were η² for ANOVA and Hedges’ g for t-tests.

### Age

A one-way ANOVA (four age groups) showed a difference in reported difficulty serving patients whose appearance did not correspond with legal identification (F(3,301) = 2.651, *p* = = 0.049). However, homogeneity of variance was violated (Levene *p* = 0.049), and Welch’s ANOVA was not statistically significant (*p* = 0.125; η² = 0.026). Bonferroni post-hoc comparisons did not identify a statistically significant pairwise difference; the comparison between the 53–62/≥63 group and the 43–52 group yielded *p* = 0.053.

### Educational level

After collapsing education to Bachelor vs Postgraduate (Master + PhD), an independent-samples *t*-test showed no significant difference between Bachelor’s and Postgraduate degree holders, t(303) = −0.10, *p* = 0.919; Welch’s t(302.7) = −0.10, *p* = 0.923. The effect size was negligible (Hedges’ g = −0.01), indicating no meaningful difference in reported difficulty by degree level.

With these group sizes, the minimum detectable effect size was approximately d = 0.32 (80% power, α = 0.05, two-tailed); the observed effect was |g|≈0.01.

### Years of professional experience

When grouped as <6 years, 6–10 years, and >10 years, professional-experience groups did not differ significantly in reported difficulties (F(2,302) = 0.401, *p* = 0.670; Welch *p* = 0.673).

### Gender

An independent-samples t-test showed no statistically significant gender difference in reported difficulties (t(303) = 0.755, *p* = 0.451; Welch’s t(294.9) = 0.755, *p* = 0.451). The effect size was Hedges’ g = 0.09 (95% CI [−0.14, 0.31]). With these group sizes, the minimum detectable effect size was approximately d = 0.32 (80% power, α = 0.05).

- Group Differences in Advocacy Participation by Demographic and Professional Factors

### Age

Advocacy and awareness participation differed significantly across the four age groups (F(3,301) = 7.058, *p* < 0.001; η² = 0.066). Heteroscedasticity was present (Levene’s *p* < 0.001), and Welch’s ANOVA also yielded *p* < 0.001. Bonferroni post-hoc tests showed that pharmacists aged 23–32 and 33–42 years reported significantly higher participation scores than the oldest combined group (53–62/≥63 years; *p* < 0.001).

### Educational level

After collapsing education categories to Bachelor versus Postgraduate, no statistically significant difference in advocacy participation was observed (t(303) = −0.293, *p* = 0.770; Welch’s t = −0.293, *p* = 0.770; Hedges’ g = −0.04, 95% CI [−0.28, 0.21]). With these group sizes, the minimum detectable effect size was approximately d = 0.35 at 80% power.

### Years of professional experience

Advocacy participation differed significantly across professional-experience groups (F(2,302) = 9.146, *p* < 0.001; η² = 0.057; Welch’s *p* < 0.001). Bonferroni comparisons showed higher participation scores among pharmacists with <6 years (MD = 0.444, *p* = 0.001) and 6–10 years (MD = 0.497, *p* = 0.003) of experience than among those with >10 years; the two groups with ≤10 years did not differ (*p* = 1.000).

Female pharmacists reported significantly higher advocacy participation scores than male pharmacists (t(303) = −4.089, *p* < 0.001; Welch’s t  = −4.123, *p* < 0.001; Hedges’ g = −0.47, 95% CI [−0.69, −0.24]). The negative sign reflects the coding used (2 = female). The minimum detectable effect size was approximately d = 0.32 at 80% power.

## Discussion

This study provides a detailed examination of the self-reported knowledge and awareness, attitudes, professional experiences, educational exposure, and educational needs regarding LGBTQ+ healthcare among community pharmacists surveyed in Thessaloniki. Although participants generally reported positive attitudes towards LGBTQ+ patients, substantial educational gaps and limited formal training were identified.

The response rate was 59.8% of the 510 pharmacists invited. Because participation was voluntary and information on nonrespondents was unavailable, the findings should be interpreted as describing the participating community pharmacists in Thessaloniki and should not be generalised to all community pharmacists in Greece. The near-equal gender distribution reflects the study sample rather than the wider pharmacist population.

Most of the participating pharmacists recognised LGBTQ+ terminology and understood distinctions between sexual orientation and gender identity, aligning with findings from prior international studies (Arthur et al., 2021; Heredia et al., 2021). However, while familiarity with basic LGBTQ+ concepts was high, only 43% of pharmacists reported sufficient education on LGBTQ+-specific healthcare needs, and just 45.9% felt fully equipped to offer appropriate care. These findings are consistent with international evidence that pharmacy education has not yet sufficiently incorporated LGBTQ+-specific healthcare into core curricula (Wahlen et al., 2020; Wilkerson et al., 2011).

Encouragingly, most pharmacists expressed a willingness to engage in further LGBTQ+-related education, with over 68% stating a clear desire for additional training, echoing similar positive intentions observed in pharmacy studies globally (Grundmann et al., 2020; Wilby et al., 2020). This willingness provides an important opportunity for pharmacy education providers, professional associations, and regulatory bodies to design and implement competency-based, inclusive training frameworks addressing LGBTQ+ patient care.

In terms of clinical behaviours, two-thirds of participants regularly served LGBTQ+ patients, and three-quarters reported no discomfort. However, over half (51.5%) experienced administrative/procedural difficulties when serving transgender and gender-diverse individuals – highlighting systemic barriers in pharmacy practice. Addressing these challenges requires not only pharmacist training but also organisational and policy-level reforms to support inclusive administrative processes.

Ethical perceptions revealed both progress and ongoing misconceptions. While 80.6% recognised the importance of protecting LGBTQ+ patient anonymity, a substantial proportion (69.1%) expressed conditional acceptance of LGBTQ+ individuals, contingent on the absence of perceived ‘provocative’ behaviours. These nuanced views may reflect deeply ingrained societal norms, highlighting the continued influence of cultural attitudes on healthcare provider biases. Similarly, misconceptions about elevated HIV risk and blood donation restrictions for gay men persist among many pharmacists, despite evolving international guidelines challenging such generalisations (CDC, 2019; WHO, 2022). These findings underscore the persistence of culturally embedded misconceptions, which may be resistant to change without targeted educational interventions. Future research using qualitative or mixed-methods approaches could further illuminate how these societal norms influence clinical practice.

A notable finding was that advocacy and awareness participation differed across age, gender, and professional-experience groups. Younger pharmacists, female pharmacists, and pharmacists with fewer years of professional experience reported higher advocacy participation. These group differences are broadly consistent with studies suggesting that younger healthcare professionals may have had more recent exposure to LGBTQ+ issues in education and society (Heredia et al., 2021; Moagi et al., 2021). The present unadjusted comparisons, however, do not establish independent demographic effects or causal influence.

In contrast, the selected self-reported difficulty outcome did not show robust differences across the demographic and professional groups examined. No statistically significant differences were identified by gender or professional experience, and the nominal age-group difference in the conventional ANOVA was not retained in the Welch analysis after the homogeneity assumption was violated. These findings distinguish the observed subgroup differences in advocacy participation from the reported difficulty outcome.

Years of professional experience did not show statistically significant group differences in the selected reported-difficulty outcome. This finding suggests that, within this sample, perceived difficulty on the analysed item was relatively similar across the three experience groups. Broader continuing education may therefore be relevant across experience levels, although the cross-sectional, unadjusted analyses do not identify which groups would benefit most.

Educational level, collapsed as Bachelor versus Postgraduate, did not show statistically significant group differences in either the selected reported-difficulty outcome or advocacy participation. This finding is compatible with calls for LGBTQ+-inclusive learning outcomes across pharmacy education and continuing professional development (FIP-EquityRx Collection 2020, n.d.; International Pharmaceutical Federation (FIP), 2020; Arthur et al., 2021), but it should not be interpreted as evidence that educational level independently predicts readiness.

Importantly, this study aligns directly with several International Pharmaceutical Federation (FIP) Development Goals. Specifically, FIP Goal 10 (Equity and Equality) is addressed by highlighting gaps in inclusive pharmacy practice and identifying areas where educational reform can foster equitable healthcare access for LGBTQ+ communities. Additionally, the study supports FIP Goal 8 (Working with Others) by emphasising the need for interprofessional, cross-sectoral, and community-based efforts to enhance LGBTQ+ health service delivery. As pharmacists often serve as first-line healthcare providers, improving their competence in LGBTQ+ care holds the potential to reduce healthcare avoidance among LGBTQ+ patients, improve trust, and ultimately contribute to better health outcomes (Fradelos et al., 2022; Grundmann et al., 2020).

### Methodological considerations

Although the sample size was adequate for detecting main effects, some subgroup analyses (e.g. by gender and experience) may have been underpowered, which should be considered when interpreting non-significant findings.

### Limitations

This cross-sectional study cannot establish causality, and its restriction to community pharmacists surveyed in Thessaloniki limits geographical generalisability; the findings should not be generalised to all community pharmacists in Greece. Recruitment was based on pharmacists accessible through the available recruitment channels, and participation was voluntary. Although 305 of the 510 invited pharmacists participated (59.8%), information on nonrespondents was unavailable; therefore, nonresponse and self-selection bias cannot be excluded. Pharmacists with greater interest in or comfort with LGBTQ+ healthcare may have been more likely to participate, whereas pharmacists who viewed the topic as sensitive or held different attitudes may have been less likely to respond. The direction and magnitude of any such bias cannot be determined. Some subgroup categories were small (e.g. Doctorate: *n* = 8; ≥63 years: *n* = 20; <1 year experience: *n* = 22), reducing power to detect small effects.

Self-reported responses may have been affected by social desirability because of the sensitivity of LGBTQ+ topics. Anonymous administration may have reduced socially desirable responding but could not eliminate it; participants may have overreported favourable attitudes or underreported discomfort and stigmatising behaviours. Social desirability was not measured using a dedicated instrument. The absence of qualitative data also limits insight into participants’ experiences and perceived barriers to inclusive care. Measures were implemented to minimise duplicate responses across online and paper surveys.

Despite these limitations, the study provides context-specific evidence from participating community pharmacists in Thessaloniki concerning LGBTQ+-related educational exposure, attitudes, professional experiences, and advocacy engagement.

## Conclusion

Among the community pharmacists surveyed in Thessaloniki, attitudes toward LGBTQ+ patients were generally positive, but important gaps in LGBTQ+-specific education and perceived preparedness remained. Advocacy and awareness participation differed across age, gender, and professional-experience groups, whereas the selected reported-difficulty outcome showed no robust demographic or professional group differences. Educational level was not significantly related to the analysed difficulty or advocacy outcomes in the unadjusted comparisons.

These findings support the inclusion of structured LGBTQ+-inclusive content in undergraduate pharmacy education and continuing professional development. Broader studies using probability-based recruitment and formally validated measurement instruments are needed to assess whether these findings extend beyond the participating pharmacists and setting studied.

## Supplementary Material

STROBE TANIS 2026.docx

FINAL TANIS 2026 Supplementary Survey S1 Questionnaire.docx
