# Supplementary material for: Community pharmacists’ knowledge, attitudes, and educational needs regarding LGBTQ+ healthcare: a cross-sectional study
Source: J Pharm Policy Pract. 2026 Nov 4;19(1):2753953. doi: 10.1080/20523211.2026.2753953 (PMC13757916; doi:10.1080/20523211.2026.2753953)
Supplement: Supplementary file 1 [file JPPP_A_2753953_SM9777.docx]

**APPENDIX 1: SURVEY QUESTIONNAIRE FOR COMMUNITY PHARMACISTS**

You are invited to participate in a study conducted by the Pharmacy Programme of the University of Nicosia regarding how community pharmacists can provide optimal support and care to LGBTQ+ individuals. Data collection will be carried out exclusively for research purposes. Completing the questionnaire does not exceed 5 minutes and the information you provide is confidential and will be used only for research purposes. By completing the questionnaire, you provide your consent to participate in the study. Your participation is voluntary and anonymous.

Thank you in advance for your participation.

ILIAS–PANAGIOTIS TANIS

**Contact details:**

Student’s e-mail: tanis.e@live.unic.ac.cy

Supervisor’s e-mail: peletidi.a@live.unic.ac.cy

**A. Understanding and Awareness**

1. The LGBTQ+ community refers to people with diverse sexual orientation, gender identity and expression.

- a. Strongly Disagree
- b. Disagree
- c. Neither Agree nor Disagree
- d. Agree
- e. Strongly Agree

2. The difference between sexual orientation and gender identity is clear to me.

- a. Strongly Disagree
- b. Disagree
- c. Neither Agree nor Disagree
- d. Agree
- e. Strongly Agree

3. I have sufficient knowledge of LGBTQ+ terminology such as “transgender”, “queer”, and “intersex”.

- a. Strongly Disagree
- b. Disagree
- c. Neither Agree nor Disagree
- d. Agree
- e. Strongly Agree

4. I have adequate education/training regarding the particular needs and concerns of LGBTQ+ individuals.

- a. Strongly Disagree
- b. Disagree
- c. Neither Agree nor Disagree
- d. Agree
- e. Strongly Agree

5. The education I received adequately covers understanding of discrimination and the needs of the LGBTQ+ community.

- a. Strongly Disagree
- b. Disagree
- c. Neither Agree nor Disagree
- d. Agree
- e. Strongly Agree

6. I feel fully equipped to provide LGBTQ+ individuals with the services and support they need.

- a. Strongly Disagree
- b. Disagree
- c. Neither Agree nor Disagree
- d. Agree
- e. Strongly Agree

7. I would like to receive more education/information about my role in caring for LGBTQ+ individuals.

- a. Strongly Disagree
- b. Disagree
- c. Neither Agree nor Disagree
- d. Agree
- e. Strongly Agree

8. I am willing to attend additional training to improve the quality of care I provide to LGBTQ+ individuals.

- a. Strongly Disagree
- b. Disagree
- c. Neither Agree nor Disagree
- d. Agree
- e. Strongly Agree

9. I would feel comfortable if my colleagues knew that I provide care to LGBTQ+ individuals.

- a. Strongly Disagree
- b. Disagree
- c. Neither Agree nor Disagree
- d. Agree
- e. Strongly Agree

**B. Ethical issues and perceptions regarding LGBTQ+ health**

10. Protecting the privacy and anonymity of LGBTQ+ individuals is essential.

- a. Strongly Disagree
- b. Disagree
- c. Neither Agree nor Disagree
- d. Agree
- e. Strongly Agree

11. I have no problem with LGBTQ+ individuals, as long as they are not “provocative” in their expressions.

- a. Strongly Disagree
- b. Disagree
- c. Neither Agree nor Disagree
- d. Agree
- e. Strongly Agree

12. I find it difficult to serve individuals whose appearance does not correspond to the details of their legal identity.

- a. Strongly Disagree
- b. Disagree
- c. Neither Agree nor Disagree
- d. Agree
- e. Strongly Agree

13. LGBTQ+ adolescents have the same health needs as non-LGBTQ+ adolescents.

- a. Strongly Disagree
- b. Disagree
- c. Neither Agree nor Disagree
- d. Agree
- e. Strongly Agree

14. Gay and lesbian individuals have higher rates of anxiety and depression compared with heterosexual individuals.

- a. Strongly Disagree
- b. Disagree
- c. Neither Agree nor Disagree
- d. Agree
- e. Strongly Agree

15. Safeguarding the anonymity of LGBTQ+ patients is vital for building trust.

- a. Strongly Disagree
- b. Disagree
- c. Neither Agree nor Disagree
- d. Agree
- e. Strongly Agree

16. Creating an open and accepting environment in the pharmacy helps LGBTQ+ individuals feel comfortable.

- a. Strongly Disagree
- b. Disagree
- c. Neither Agree nor Disagree
- d. Agree
- e. Strongly Agree

17. For public health reasons, donor safety must be considered; gay men should not be allowed to donate blood because they are at higher risk for HIV.

- a. Strongly Disagree
- b. Disagree
- c. Neither Agree nor Disagree
- d. Agree
- e. Strongly Agree

18. LGBTQ+ individuals are more likely to contract HIV.

- a. Strongly Disagree
- b. Disagree
- c. Neither Agree nor Disagree
- d. Agree
- e. Strongly Agree

**C. Attitudes and Behaviours**

19. I serve LGBTQ+ individuals.

- a. Never
- b. Rarely
- c. Sometimes
- d. Often
- e. Always

20. I have felt discomfort when serving LGBTQ+ individuals.

- a. Never
- b. Rarely
- c. Sometimes
- d. Often
- e. Always

21. I have faced difficulties in serving LGBTQ+ individuals due to administrative/procedural barriers (e.g., identity verification, paperwork) that may discourage safe and anonymous access to pharmacy services.

- a. Never
- b. Rarely
- c. Sometimes
- d. Often
- e. Always

22. I find it difficult to serve a person whose appearance does not match the details of their identification.

- a. Never
- b. Rarely
- c. Sometimes
- d. Often
- e. Always

23. At my workplace I have discussed issues of sexual orientation and/or gender identity.

- a. Never
- b. Rarely
- c. Sometimes
- d. Often
- e. Always

24. My colleagues make “harmless” jokes or comments about LGBTQ+ individuals.

- a. Never
- b. Rarely
- c. Sometimes
- d. Often
- e. Always

25. I make jokes with colleagues about LGBTQ+ individuals.

- a. Never
- b. Rarely
- c. Sometimes
- d. Often
- e. Always

**D. Active support and awareness**

26. It has happened that a colleague of mine insulted or abused a person because of sexual orientation and/or gender identity.

- a. Never
- b. Rarely
- c. Sometimes
- d. Often
- e. Always

27. I have participated in events or initiatives in favour of the rights of LGBTQ+ individuals.

- a. Never
- b. Rarely
- c. Sometimes
- d. Often
- e. Always

28. I have implemented actions to promote understanding and awareness of the needs of LGBTQ+ individuals in my community.

- a. Never
- b. Rarely
- c. Sometimes
- d. Often
- e. Always

29. I have used information sources on LGBTQ+ issues from literature and scientific articles.

- a. Never
- b. Rarely
- c. Sometimes
- d. Often
- e. Always

**E. Demographic characteristics**

30. Age

- a. 23–32
- b. 33–42
- c. 43–52
- d. 53–62
- e. 63 and over

31. Gender

- a. Male
- b. Female
- c. Other

32. Education as a pharmacist

- a. Bachelor’s degree
- b. Master’s degree
- c. Doctorate

33. Position in the pharmacy

- a. Employee
- b. Owner

34. Experience as a pharmacist

- a. Less than 1 year
- b. 1–5 years
- c. 6–10 years
- d. More than 10 years

35. Do you self-identify as an LGBTQ+ person?

- a. Yes
- b. No
- c. I prefer not to answer
