# Supplementary material for: Community pharmacists’ knowledge, attitudes, and educational needs regarding LGBTQ+ healthcare: a cross-sectional study
Source: J Pharm Policy Pract. 2026 Nov 4;19(1):2753953. doi: 10.1080/20523211.2026.2753953 (PMC13757916; doi:10.1080/20523211.2026.2753953)
Supplement: Supplementary file 2 [file JPPP_A_2753953_SM9778.docx]

**STROBE Statement – Checklist of items that should be included in reports of cross-sectional studies**

| **Item No.** | **Section** | **Recommendation** | **How addressed** |
| --- | --- | --- | --- |
| **Title and Abstract** |  |  |  |
| 1a | Title | Indicate the study’s design with a commonly used term | Title includes: "A Cross-Sectional Study..." |
| 1b | Abstract | Provide informative and balanced summary of what was done and found | Structured abstract provided (Background, Objectives, Methods, Results, Conclusion) |
| **Introduction** |  |  |  |
| 2 | Background/rationale | Explain scientific background and rationale | Explained in Introduction with relevant references |
| 3 | Objectives | State specific objectives, including any prespecified hypotheses | Clearly stated at end of Introduction |
| **Methods** |  |  |  |
| 4 | Study design | Present key elements of study design early | Design described as cross-sectional in Methods |
| 5 | Setting | Describe setting, locations, dates | Thessaloniki, Greece, November 2023 |
| 6a | Participants | Eligibility criteria and selection methods | All actively practising community pharmacists in Thessaloniki invited |
| 7 | Variables | Clearly define outcomes, exposures, predictors | Described in "Variables" section |
| 8 | Data sources/measurement | Describe data sources and assessment methods | Validated 35-item questionnaire used (described in Methods) |
| 9 | Bias | Describe efforts to address bias | Anonymity emphasised to reduce social desirability bias |
| 10 | Study size | Explain how study size was arrived at | Based on total pharmacist population and targeted response rate |
| 11 | Quantitative variables | Explain how variables handled and grouped | Descriptive and inferential statistics detailed |
| 12a | Statistical methods | Describe all statistical methods used | T-tests, ANOVA, descriptive statistics described |
| 12b | Subgroups/interactions | Describe any subgroup analysis | Subgroups analysed for age, gender, education, experience |
| 12c | Missing data | Explain missing data handling | Not applicable (complete dataset) |
| 12d | Sampling strategy | Describe if applicable | Census-based recruitment of local pharmacist population |
| 12e | Sensitivity analyses | Describe if done | Not applicable |
| **Results** |  |  |  |
| 13a | Participants | Report numbers at each stage | Total invited and response rate reported |
| 13b | Non-participation | Report reasons for non-participation | Not reported; voluntary anonymous survey |
| 13c | Flow diagram | Use if applicable | Not used |
| 14a | Descriptive data | Participant characteristics | Full demographic table provided (Table 1) |
| 14b | Missing data | Report number of missing data for each variable | Not applicable (complete data) |
| 15 | Outcome data | Report outcome events or summary measures | Fully reported for all questionnaire items (Tables 2–6) |
| 16a | Main results | Unadjusted and adjusted estimates provided | Fully reported with p-values and test statistics |
| 16b | Category boundaries | Report cut-off values where applicable | Not applicable (Likert-scale variables) |
| 16c | Relative risks | Translate estimates into risks if applicable | Not applicable |
| 17 | Other analyses | Report subgroup and interaction analyses | Full inferential statistics presented (ANOVA, t-tests) |
| **Discussion** |  |  |  |
| 18 | Key results | Summarise key results | Fully summarised with reference to objectives |
| 19 | Limitations | Discuss study limitations | Addressed in Discussion |
| 20 | Interpretation | Interpret considering limitations and literature | Fully discussed with supporting citations |
| 21 | Generalisability | Discuss generalisability | Addressed in Discussion |
| **Other Information** |  |  |  |
| 22 | Funding | Describe funding and role of funders | No external funding received |
